# Supplementary material for: Estimating psychological networks and their accuracy: A tutorial paper
Source: Behav Res Methods. 2017 Mar 24;50(1):195–212. doi: 10.3758/s13428-017-0862-1 (PMC5809547; doi:10.3758/s13428-017-0862-1)
Supplement: Supplementary file 1 — (PDF 218 KB) [file 13428_2017_862_MOESM1_ESM.pdf]

Estimating Psychological Networks and their Accuracy: Supplementary Materials

Sacha Epskamp, Denny Borsboom and Eiko I. Fried
Department of Psychology, University of Amsterdam

Contents

Psychological Networks 1
Directed and Undirected Networks . . . 2
Network Inference . . . 3

The bootstrap and centrality indices 4

Loading the PTSD dataset 6

Psychological Networks

A psychological network is a model in which *nodes* represent observed psychological variables, usually psychometric test items such as responses to questions about whether a person suffered from insomnia or fatigue in past weeks. These nodes are connected by *edges* which indicate some statistical relationship between them. These models are conceptually different from commonly used reflective latent variable models that explain the co-occurrence among symptoms (e.g., the fact that individuals often suffer from sadness, insomnia, fatigue, and concentration problems at the same time) by invoking an underlying unobserved latent trait (e.g., depression) as the common cause of all the symptoms. Psychological networks offer a different conceptual interpretation of the data and explain such co-occurrences via direct relationships between symptoms; for example, someone who sleeps poorly will be tired, and someone who is tired will not concentrate well (Fried et al., 2015; Schmittmann et al., 2013). Such relationships can then be more easily interpreted when drawn as a network structure where edges indicate pathways on which nodes can affect each other. The edges can differ in strength of connection, also termed *edge weight* (Epskamp, Cramer, Waldorp, Schmittmann, & Borsboom, 2012), indicating if a relationship is strong (commonly visualized with thick edges) or weak (thin, less saturated edges) and positive (green edges) or negative (red edges). After a network structure is estimated, the visualization of the graph itself tells the researcher a detailed story of the multivariate dependencies in the data. Additionally, many inference methods from graph theory can be used to assess which nodes are the most important in the network, termed the *most central nodes.*

Directed and Undirected Networks

In general, there are two types of edges that can be present in a network: an edge can be directed, in which case one head of the edge has an arrowhead indicating a one-way effect, or an edge can be undirected, indicating some mutual relationship. A network that contains only directed edges is termed a *directed network*, whereas a network that contains only undirected edges is termed an *undirected network* (Newman, 2010). Many fields of science consider directed networks interesting because they can be used to encode causal structures (Pearl, 2000). For example, the edge insomnia → fatigue can be taken to indicate that insomnia causes fatigue. The work of Pearl describes that such causal structures can be tested using only observational cross-sectional data and can even be estimated to a certain extent (Kalisch, Mächler, Colombo, Maathuis, & Bühlmann, 2012; Scutari, 2010). However, when temporal information is lacking, there is only limited information present in cross-sectional observational data. Such estimation methods typically only work under two very strict assumptions (a) that all entities which play a causal role have been measured and (b) that the causal chain of cause and effect is not cyclic (i.e., a variable cannot cause itself via any path). Both assumptions are not very plausible in psychological systems. Furthermore, such directed networks suffer from the problem that many equivalent models can exist that feature the same relationships found in the data (MacCallum, Wegener, Uchino, & Fabrigar, 1993); this makes the interpretation of structures difficult. For example, the structure insomnia → fatigue → concentration is statistically equivalent to the structure insomnia ← fatigue → concentration as well as the structure insomnia ← fatigue ← concentration: All three only indicate that insomnia and concentration problems are conditionally independent after controlling for fatigue.

For the reasons outlined above, psychological networks estimated on cross-sectional data are typically undirected networks. The current state-of-the-art method for estimating undirected psychological network structures involves the estimation of PMRFs. A PMRF is a network model in which edges indicate the full conditional association between two nodes after conditioning on all other nodes in the network. This means when two nodes are connected, there is a relationship between these two nodes that cannot be explained by any other node in the network. Simplified, it can be understood as a partial correlation controlling for all other connections. The absence of an edge between two nodes indicates that these nodes are conditionally independent of each other given the other nodes in the network. Thus, a completely equivalent undirected structure (compared to the structures described above) would be insomnia — fatigue — concentration, indicating that insomnia and concentration problems are conditionally independent after controlling for fatigue.

Figure 1 shows a PMRF similar to the example described above. In this network, there is a positive relationship between insomnia and fatigue and a negative relationship between fatigue and concentration. The positive edge is thicker and more saturated than the negative edge, indicating that this interaction effect is stronger than that of the negative edge. This network shows that insomnia and concentration do not directly interact with each other in any way other than through their common connection with fatigue. Therefore, fatigue is the most important node in this network—a concept we will later quantify as *centrality*. These edges can be interpreted in several different ways. First, as shown above, the model is in line with causal interpretations of associations among the symptoms. Second, this model implies

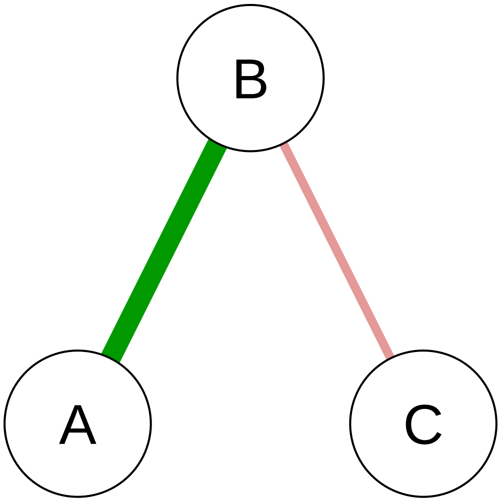

Figure 1. Example of a pairwise Markov random field. Node A positively interacts with node B, and node B negatively interacts with node C. Nodes A and C are *conditionally independent* given node B.

that insomnia and fatigue predict each other after controlling for concentration; even when we know someone is concentrating poorly, that person is more likely to suffer from insomnia when we observe that person suffering from fatigue. Similarly, fatigue and concentration predict each other after controlling for insomnia. After controlling for fatigue, there is no longer any predictive quality between insomnia and concentration, even though these variables are correlated; fatigue now mediates the prediction between these two symptoms (Epskamp, Maris, Waldorp, & Borsboom, in press). Finally, these edges can represent genuine symmetric causal interactions between symptoms (e.g., in statistical physics, a PRMF called the Ising model is used to model particles that cause neighboring particles to be aligned). We refer the reader to Epskamp, Waldorp, Mõttus, and Borsboom for a longer introduction to the above-mentioned interpretation of edges in a PMRF.

Network Inference

In the first step of network analysis, the obtained network is typically presented graphically to show the structure of the data (Epskamp et al., 2012). Afterwards, inference methods derived from graph theory can be applied to the network structure. The estimated PRMF is always a weighted network, which means that we not only look at the structure of the network (e.g., are two nodes connected or not) but also at the strength of connection between pairs of nodes. Because of this, many typical inference methods that concern the global structure of the network (e.g., small-worldness, density, and global clustering; Kolaczyk, 2009; Newman, 2010; Watts and Strogatz, 1998) are less useful in the context of

psychological networks because they only take into account whether nodes are connected or not and not the strength of association among nodes. Because the global inference methods for weighted networks and PRMFs are still in development and no consensus has been reached, the network inference section focuses on local network properties: How are two nodes related, and what is the influence of a single node?

Relationship between two nodes. The relationship between two nodes can be assessed in two ways. First, we can directly assess the edge weight. This is always a number that is nonzero because an edge weight of zero would indicate there is no edge. The sign of the edge weight (positive or negative) indicates the type of interaction, and the absolute value of the edge weight indicates the strength of the effect. For example, a positive edge weight of 0.5 is equal in strength to a negative edge weight of -0.5 and both are stronger than an edge weight of 0.2. Two strongly connected nodes influence each other more easily than two weakly connected nodes. This is similar to how two persons standing closer to each other can communicate more easily (via talking) than two people standing far away from each other (via shouting)—two strongly connected nodes are closer to each other. As such, the length of an edge is defined as the inverse of the edge strength. Finally, the distance between two nodes is equal to the sum of the lengths of all edges on the shortest path between two nodes (Newman, 2010).

Node centrality. The importance of individual nodes in the network can be assessed by investigating the node centrality. A visualization of a network, such as the one shown in Figure 1, is an abstract rendition of a high-dimensional space in two dimensions. Although visualizations of network models often aim to place highly connected nodes into the center of the graph, for instance using the Fruchterman-Reingold algorithm (Fruchterman & Reingold, 1991), the two-dimensional visualization cannot properly reflect the true space of the model. Thus, the metric distance between the placement of nodes in the two-dimensional space has no direct interpretation as it has in multidimensional scaling, for instance. Therefore, graph theory has developed several methods to more objectively quantify which node is most central in a network. Three such centrality measures have appropriate weighted generalizations that can be used with psychological networks (Opsahl, Agneessens, & Skvoretz, 2010). First, node strength, also called degree in unweighted networks (Newman, 2010), simply adds up the strength of all connected edges to a node; if the network is made up of partial correlation coefficients, the node strength equals the sum of absolute partial correlation coefficients between a node and all other nodes. Second, closeness takes the inverse of the sum of all the shortest paths between one node and all other nodes in the network. Thus, where node strength investigates how strongly a node is directly connected to other nodes in the network, closeness investigates how strongly a node is indirectly connected to other nodes in the network. Finally, betweenness looks at how many of the shortest paths between two nodes go through the node in question; the higher the betweenness, the more important a node is in connecting other nodes.

The bootstrap and centrality indices

The bootstrap is not a foolproof methodology that always works. There are, in fact, many cases in which the bootstrap fails (Chernick, 2011). One such case is when the parameter of interest has an expected value at the boundary of the parameter space. In bootstrapping edge-weights (as described in the main manuscript above), such as partial

correlation coefficients, this is not a problem because edge-weights at the boundary of 1 or -1 rarely occur. However, when continuing network inference, we do not use the edge-weights directly but instead the edge strength: the absolute value of the edge-weight. Here, a boundary problem *is* present, as we expect many edges to have an expected value of exactly zero (implying the absence of an edge). To exemplify, suppose we find an edge-weight of 0.01 in our original sample, with a standard error of 0.1. When bootstrapping, 95% of the samples should fall roughly in the interval -0.19 to 0.21; the absolute value of these samples will, almost always, be *higher* than the original sampled edge strength.

We discovered a *two-sided* bias when many edge-weights are expected to equal zero (sparse network structure). First, absolute edge-weights, and as a result centrality indices, are highly biased estimators of the true parameter. This makes sense: if the true edge-weight is zero, any estimated absolute edge-weight based on a sample would be higher than zero. Second, bootstrapped absolute edge-weights are biased as well when the expected value is zero, because the bootstrap sampling distribution is likely to be centered near zero leading to many higher absolute edge-weights. To exemplify, we simulated 10,000 pairs of independent variables (true correlation of zero) and investigated the sample absolute correlation as well as the absolute correlation of a single nonparametric and parametric bootstrap. The sample correlation was on average 0.081 higher than the true correlation of zero, the nonparametric bootstrap was on average 0.032 higher than the sampled absolute correlation and the parametric bootstrap was on average 0.033 higher than the sampled absolute correlation.

Computing node strength takes a sum and computing closeness takes the inverse of a more complicated sum of these biased values; adding up these biases leads to highly inaccurate estimates of the true parameter. Indeed, we found that it is not uncommon for quantiles of the bootstrapped strength and closeness to *not* contain the true parameter at all, nor even the parameter value based on the original sample. This bias seemed to be mostly present using non-regularization estimation methods and low sample sizes. Thus, constructing true CIs for centrality indices—containing the true parameter 95% of the times—is a challenging research question, involving correcting for bias of the sample due to true network structure and sample size as well as correcting for the bias in the bootstrap samples. The current paper does not aim to solve this highly technical question, as we are not so much interested in estimating a region around the true parameter value as that we are interested in showing the sampling variability of these centrality indices, even if they are biased.

As explained above, the bootstrap cannot be used to form 95% CIs on centrality indices. However, we can use the bootstrap to show the reader the importance of taking centrality in accuracy into account. If we assume that the estimated partial correlation matrix is the true network structure, then the parametric bootstrap can be used to show the sampling distribution of such a network model:

```
boot3 <- bootnet(Data, 100, default = "pcor", type = "parametric")
```

The 95% quantile regions can be plotted using the following codes:

```
plot(boot3, statistics = c("strength", "closeness", "betweenness"),
CIstyle = "quantiles")
```

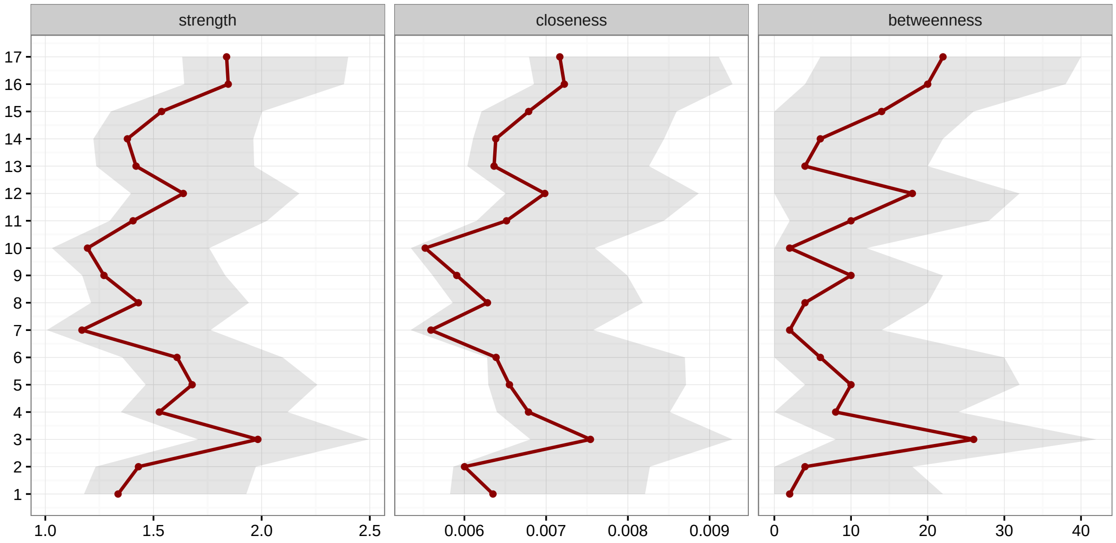

Figure 2. Parametric bootstrap on centrality indices based on the partial correlation network.

which will return a warning on that the intervals cannot be interpreted as 95% CIs. The argument `CIstyle = "quantiles"` makes sure quantiles are plotted. By default, bootnet plots intervals based on the sample centrality plus and minus two times the standard deviation of the bootstraps. Which are more interpretable, even though they still are not true 95% CIs.

Figure 2 shows the resulting plot. If the partial correlation network used is the true model, then the red line shows the true centrality indices and the gray area the 95% quantile region of the sampling distribution. The placement of the red line indicates the aforementioned bias in both bootstrapping and sampling of centrality indices. The size of the gray area is large in all indices, indicating that the order of centrality indices from any sample would be very hard to interpret. In the sample on which these plots are based, none of the 1000 samples captured the true order of any of the centrality indices, and the probability that the node with the true strongest centrality was correctly detected was only 43%, 36% and 33% for strength, closeness and betweenness respectively.

These plots only show the sampling distribution if the used network is the true model—which it is not (as the network is estimated from data). Therefore, these plots should be interpreted with care. However, they do highlight the importance of accuracy analysis on centrality indices, as the sampling distribution of the true model is likely not much smaller than the ones shown in these plots.

Loading the PTSD dataset

To download the dataset, go to:

https://datashare.nida.nih.gov/study/nida-ctn-0015

and click on "CTN-0015 Data Files". The relevant data file is called "qs.csv", which can be loaded into R by using the default `read.csv` function:

```
FullData <- read.csv("qs.csv", stringsAsFactors = FALSE)
```

This loads the data in *long* format, which contains a column with subject id's, a column with the names of the administered items, and a third column containing the item responses. For network analysis, we need the data to be in *wide* format. Furthermore, we need to assign that the response `"NOT ANSWERED"` indicates a missing response and other responses are ordinal. Finally, we need to extract relevant dataset at baseline measure for the PTSD symptom frequency scores. To do this, we can utilize the *dplyr* and *tidyr* R packages as follows:

```
# Load packages:
library("dplyr")
library("tidyr")
# Frequency at baseline:
Data <- FullData %>%
  filter(EPOCH == "BASELINE",
         grepl("^PSSR\\d+A$",QSTESTCD)) %>%
  select(USUBJID,QSTEST,QSORRES) %>%
  spread(QSTEST, QSORRES) %>%
  select(-USUBJID) %>%
  mutate_each(funs(replace(.,.=="NOT ANSWERED",NA))) %>%
  mutate_each(funs(ordered(.,c("NOT AT ALL","ONCE A WEEK",
                             "2-4 TIMES PER WEEK/HALF THE TIME",
                             "5 OR MORE TIMES PER WEEK/ALMOST ALWAYS"))))
names(Data) <- seq_len(ncol(Data))
```

References

Chernick, M. R. (2011). *Bootstrap methods: a guide for practitioners and researchers.* New York, NY, USA: John Wiley & Sons.

Epskamp, S., Maris, G., Waldorp, L., & Borsboom, D. (in press). Network psychometrics. In P. Irwing, D. Hughes, & T. Booth (Eds.), *Handbook of psychometrics.* New York, NY, USA: Wiley.

Epskamp, S., Cramer, A., Waldorp, L., Schmittmann, V. D., & Borsboom, D. (2012). Qgraph: network visualizations of relationships in psychometric data. *Journal of Statistical Software*, *48*(1), 1–18.

Epskamp, S., Waldorp, L. J., Mõttus, R., & Borsboom, D. (2016). Discovering psychological dynamics: the gaussian graphical model in cross-sectional and time-series data. *arXiv preprint*, arXiv:1609.04156.

Fried, E. I., Bockting, C., Arjadi, R., Borsboom, D., Amshoff, M., Cramer, O. J., . . . Stroebe, M. (2015). From loss to loneliness: The relationship between bereavement and depressive symptoms. *Journal of abnormal psychology*, *124*(2), 256–265.

Fruchterman, T. & Reingold, E. (1991). Graph drawing by force-directed placement. *Software: Practice and Experience*, *21*(11), 1129–1164.

Kalisch, M., Mächler, M., Colombo, D., Maathuis, M. H., & Bühlmann, P. (2012). Causal inference using graphical models with the r package pcalg. *Journal of Statistical Software*, *47*(11), 1–26.

Kolaczyk, E. D. (2009). *Statistical analysis of network data*. New York, NY, USA: Springer.

MacCallum, R. C., Wegener, D. T., Uchino, B. N., & Fabrigar, L. R. (1993). The problem of equivalent models in applications of covariance structure analysis. *Psychological bulletin*, *114*(1), 185–199.

Newman, M. E. J. (2010). *Networks: an introduction*. Oxford, UK: Oxford University Press.

Opsahl, T., Agneessens, F., & Skvoretz, J. (2010). Node centrality in weighted networks: generalizing degree and shortest paths. *Social Networks*, *32*(3), 245–251.

Pearl, J. (2000). *Causality: Models, Reasoning, and Inference*. Cambridge Univ Pr.

Schmittmann, V. D., Cramer, A. O. J., Waldorp, L. J., Epskamp, S., Kievit, R. A., & Borsboom, D. (2013). Deconstructing the construct: A network perspective on psychological phenomena. *New Ideas in Psychology*, *31*(1), 43–53.

Scutari, M. (2010). Learning Bayesian Networks with the bnlearn R Package. *Journal of Statistical Software*, *35*(3), 1–22.

Watts, D. & Strogatz, S. (1998). Collective dynamics of small-world networks. *Nature*, *393*(6684), 440–442.
